# Supplementary material for: Antimicrobial Resistance Patterns and Risk Factors Associated with ESBL-Producing and MDR Escherichia coli in Hospital and Environmental Settings in Lusaka, Zambia: Implications for One Health, Antimicrobial Stewardship and Surveillance Systems
Source: Microorganisms. 2023 Jul 31;11(8):1951. doi: 10.3390/microorganisms11081951 (PMC10459584; doi:10.3390/microorganisms11081951)
Supplement: Supplementary file 1 [file microorganisms-11-01951-s001.zip › microorganisms-2505670-supplementary.pdf]

microorganisms

MDPI

Article

Antimicrobial Resistance Patterns and Risk Factors Associated with ESBL-Producing and MDR *Escherichia coli* in Hospital and Environmental Settings in Lusaka, Zambia: Implications for One Health, Antimicrobial Stewardship and Surveillance Systems

Table S1. Antimicrobial Susceptibility of Clinical *E. coli* categorized according to sample types.

Table S2. Antimicrobial Susceptibility of Clinical *E. coli* categorized according to hospital departments.

Table S3. Antimicrobial Susceptibility of Environmental *E. coli* categorized according to sample types.

Figure S1. Distribution of MDR and XDR environmental *E. coli* isolates categorized according to sample types.

Figure S2. Distribution of MDR and XDR clinical *E. coli* isolates categorized according to sample types.

Figure S3. Distribution of MDR and XDR clinical *E. coli* isolates categorized according to hospital departments.

Table S1. Antimicrobial Susceptibility of Clinical *E. coli* categorized according to sample types

Antibiotics		Specimen types (n, %)					Total
		Blood	CSF	Pus	Stool	Urine	(n)
CN	S	9 (75%)	9 (81.8%)	53 (88.3%)	2 (100%)	208 (84.2%)	281
	I	0 (0%)	1 (9.1%)	1 (1.7%)	0 (0%)	5 (2%)	7
	R	3 (25%)	1 (9.1%)	6 (10%)	0 (0%)	34 (13.8%)	44
ETP	S	12 (100%)	11 (100%)	56 (93.3%)	2 (100%)	245 (99.2%)	326
	I	0 (0%)	0 (0%)	2 (3.3%)	0 (0%)	2 (0.8%)	4

	R	0 (0%)	0 (0%)	2 (3.3%)	0 (0%)	0 (0%)	2
IPM	S	12 (100%)	11 (100%)	56 (93.3%)	2 (100%)	247 (100%)	328
	I	0 (0%)	0 (0%)	2 (3.3%)	0 (0%)	0 (0%)	2
	R	0 (0%)	0 (0%)	2 (3.3%)	0 (0%)	0 (0%)	2
KZ	S	0 (0%)	0 (0%)	5 (8.3%)	1 (50%)	107 (43.3%)	113
	I	0 (0%)	0 (0%)	3 (5%)	1 (50%)	0 (0%)	4
	R	12 (100%)	11 (100%)	52 (86.7%)	0 (0%)	140 (56.7%)	215
CXM	S	0 (0%)	0 (0%)	8 (13.3%)	2 (100%)	107 (43.3%)	117
	I	0 (0%)	0 (0%)	2 (3.3%)	0 (0%)	6 (2.4%)	8
	R	12 (100%)	11 (100%)	50 (83.3%)	0 (0%)	134 (54.3%)	207
CAZ	S	4 (33.3%)	1 (9.1%)	25 (41.7%)	2 (100%)	125 (50.6%)	157
	I	0 (0%)	0 (0%)	2 (3.3%)	0 (0%)	7 (2.8%)	9
	R	8 (66.7%)	10 (90.9%)	33 (55%)	0 (0%)	115 (46.6%)	166
CRO	S	0 (0%)	0 (0%)	10 (16.7%)	2 (100%)	111 (44.9%)	123
	R	12 (100%)	11 (100%)	50 (83.3%)	0 (0%)	136 (55.1%)	209
FEP	S	2 (16.7%)	1 (9.1%)	25 (41.7%)	2 (100%)	129 (52.2%)	159
	R	10 (83.3%)	10 (90.9%)	35 (58.3%)	0 (0%)	118 (47.8%)	173
AMP	S	0 (0%)	0 (0%)	5 (8.3%)	0 (0%)	50 (20.2%)	55
	R	12 (100%)	11 (100%)	55 (91.7%)	2 (100%)	197 (79.8%)	277
AMC	S	4 (33.3%)	3 (27.3%)	32 (53.3%)	0 (0%)	79 (32%)	118
	I	6 (50%)	0 (0%)	10 (16.7%)	0 (0%)	82 (33.2%)	98
	R	2 (16.7%)	8 (72.7%)	18 (30%)	2 (100%)	86 (34.8%)	116
SXT	S	0 (0%)	2 (18.2%)	10 (16.7%)	2 (100%)	73 (29.6%)	87
	R	12 (100%)	9 (81.8%)	50 (83.3%)	0 (0%)	174 (70.4%)	245
NIT	S	10 (83.3%)	11 (100%)	58 (96.7%)	2 (100%)	217 (87.9%)	298
	I	0 (0%)	0 (0%)	0 (0%)	0 (0%)	18 (7.3%)	18
	R	2 (16.7%)	0 (0%)	2 (3.3%)	0 (0%)	12 (4.9%)	16
CIP	S	2 (16.7%)	0 (0%)	18 (30%)	2 (100%)	91 (36.8%)	113
	I	0 (0%)	0 (0%)	0 (0%)	0 (0%)	1 (0.4%)	1
	R	10 (83.3%)	11 (100%)	42 (70%)	0 (0%)	155 (62.8%)	218
LEV	S	2 (16.7%)	0 (0%)	17 (28.3%)	2 (100%)	98 (39.7%)	119
	R	10 (83.3%)	11 (100%)	43 (71.7%)	0 (0%)	149 (60.3%)	213

Abbreviations: AMC-Amoxicillin/clavulanic acid; FEP-Cefepime; CN-Gentamicin; AMP- Ampicillin; IMP-Imipenem; CAZ-Ceftazidime; KZ-Cephazolin; CIP-Ciprofloxacin; LEV-Levofloxacin; NIT-Nitrofurantoin; CRO-Ceftriaxone; CXM-Cefuroxime; SXT-Sulfamethoxazole/trimethoprim; ETP-Ertapenem

Table S2. Antimicrobial Susceptibility of Clinical *E. coli* categorized according to hospital departments

**Antibiotics**	**Hospital departments (n, %)**							**Total (n)**
	**Admission**	**General adult**	**Intensive care unit**	**OBGYN**	**Outpatient department**	**Pediatrics and neonatology**	**Surgery**	

CN	S	7 (87.5%)	46 (88.5%)	15 (71.4%)	34 (89.5%)	95 (80.5%)	43 (95.6%)	41 (82%)	281
	R	1 (12.5%)	6 (11.5%)	6 (28.6%)	3 (7.9%)	19 (16.1%)	1 (2.2%)	8 (16%)	44
ETP	S	8 (100%)	52 (100%)	19 (90.5%)	38 (100%)	115 (97.5%)	45 (100%)	49 (98%)	326
	R	0 (0%)	0 (0%)	0 (0%)	0 (0%)	1 (0.8%)	0 (0%)	1 (2%)	2
IPM	S	8 (100%)	52 (100%)	21 (100%)	36 (94.7%)	117 (99.2%)	45 (100%)	49 (98%)	328
	R	0 (0%)	0 (0%)	0 (0%)	0 (0%)	1 (0.8%)	0 (0%)	1 (2%)	2
KZ	S	6 (75%)	13 (25%)	3 (14.3%)	15 (39.5%)	53 (44.9%)	12 (26.7%)	11 (22%)	113
	R	2 (25%)	39 (75%)	18 (85.7%)	23 (60.5%)	64 (54.2%)	33 (73.3%)	36 (72%)	215
CXM	S	6 (75%)	14 (26.9%)	3 (14.3%)	15 (39.5%)	54 (45.8%)	12 (26.7%)	13 (26%)	117
	R	2 (25%)	37 (71.2%)	18 (85.7%)	20 (52.6%)	60 (50.8%)	33 (73.3%)	37 (74%)	207
CAZ	S	6 (75%)	21 (40.4%)	7 (33.3%)	15 (39.5%)	63 (53.4%)	21 (46.7%)	24 (48%)	157
	R	2 (25%)	29 (55.8%)	14 (66.7%)	21 (55.3%)	52 (44.1%)	22 (48.9%)	26 (52%)	166
CRO	S	6 (75%)	15 (28.8%)	3 (14.3%)	17 (44.7%)	57 (48.3%)	12 (26.7%)	13 (26%)	123
	R	2 (25%)	37 (71.2%)	18 (85.7%)	21 (55.3%)	61 (51.7%)	33 (73.3%)	37 (74%)	209
FEP	S	6 (75%)	22 (42.3%)	7 (33.3%)	19 (100%)	63 (53.4%)	20 (44.4%)	22 (44%)	159
	R	2 (25%)	30 (57.7%)	14 (66.7%)	19 (100%)	55 (46.6%)	25 (55.6%)	28 (56%)	173
AMP	S	3 (37.5%)	4 (7.7%)	2 (9.5%)	9 (23.7%)	21 (17.8%)	8 (17.8%)	8 (16%)	55
	R	5 (62.5%)	48 (92.3%)	19 (90.5%)	29 (76.3%)	97 (82.2%)	37 (82.2%)	42 (84%)	277
AMC	S	6 (75%)	11 (21.2%)	8 (38.1%)	18 (47.4%)	40 (33.9%)	11 (24.4%)	24 (48%)	118
	R	0 (0%)	18 (34.6%)	8 (38.1%)	16 (42.1%)	42 (35.6%)	19 (42.2%)	13 (26%)	116
SXT	S	4 (50%)	17 (32.7%)	3 (14.3%)	7 (18.4%)	38 (32.2%)	13 (28.9%)	5 (10%)	87
	R	4 (50%)	35 (67.3%)	18 (85.7%)	31 (81.6%)	80 (67.8%)	32 (71.1%)	45 (90%)	245
NIT	S	6 (75%)	43 (82.7%)	18 (85.7%)	36 (94.7%)	103 (87.3%)	43 (95.6%)	49 (98%)	298
	R	0 (0%)	6 (11.5%)	2 (9.5%)	0 (0%)	6 (5.1%)	1 (2.2%)	1 (2%)	16
CIP	S	5 (62.5%)	12 (23.1%)	5 (23.8%)	16 (42.1%)	43 (36.4%)	18 (40%)	14 (28%)	113
	R	3 (37.5%)	40 (76.9%)	16 (76.2%)	22 (57.9%)	74 (62.7%)	27 (60%)	36 (72%)	218
LEV	S	6 (75%)	13 (25%)	5 (23.8%)	17 (44.7%)	47 (39.8%)	17 (37.8%)	14 (28%)	119
	R	2 (25%)	39 (75%)	16 (76.2%)	21 (55.3%)	71 (60.2%)	28 (62.2%)	36 (72%)	213

Abbreviations: AMC-Amoxicillin/clavulanic acid; FEP-Cefepime; CN-Gentamicin; AMP- Ampicillin; IMP-Imipenem; CAZ-Ceftazidime; KZ-Cephazolin; CIP-Ciprofloxacin; LEV-Levofloxacin; NIT-Nitrofurantoin; CRO-Ceftriaxone; CXM-Cefuroxime; SXT-Sulfamethoxazole/trimethoprim; ETP-Ertapenem

Table S3. Antimicrobial Susceptibility of Environmental *E. coli* categorized according to sample types

**Antibiotics**		**Sample type (n, %)**						**Total (n)**
		**Chicken**	**Fish**	**Water**	**meats**	**Fruits and Vegetables**	**Hospital Equipment**	
AMP	S	7 (77.8%)	22 (75.9%)	22 (62.9%)	44 (78.6%)	44 (67.7%)	102 (96.2%)	241
	R	2 (22.2%)	7 (24.1%)	13 (37.1%)	12 (21.4%)	21 (32.3%)	4 (3.8%)	59
AMC	S	8 (88.9%)	22 (75.9%)	22 (62.5%)	52 (92.9%)	51 (78.5%)	83 (78.3%)	238
	R	0 (0%)	5 (17.2%)	12 (34.3%)	3 (5.4%)	13 (20%)	20 (18.9%)	53
KZ	S	9 (100%)	21 (72.4%)	21 (60%)	47 (83.9%)	44 (67.7%)	73 (68.9%)	215

	R	0 (0%)	8 (27.6%)	14 (40%)	9 (16.1%)	21 (32.3%)	33 (31.1%)	85
CXM	S	8 (88.9%)	23 (79.3%)	22 (62.5%)	51 (91.1%)	48 (73.8%)	83 (78.3%)	235
	I	1 (11.1%)	0 (0%)	0 (0%)	1 (1.8%)	0 (0%)	2 (1.9%)	4
	R	0 (0%)	6 (20.7%)	13 (37.1%)	4 (7.1%)	17 (26.2%)	21 (19.8%)	61
CAZ	S	9 (100%)	23 (79.3%)	23 (65.7%)	53 (94.6%)	50 (76.9%)	85 (80.2%)	243
	R	0 (0%)	6 (20.7%)	12 (34.3%)	3 (5.4%)	15 (23.1%)	21 (19.8%)	57
CRO	S	9 (100%)	23 (79.3%)	22 (62.5%)	53 (94.6%)	48 (73.8%)	86 (81.1%)	241
	R	0 (0%)	6 (20.7%)	13 (37.1%)	3 (5.4%)	17 (26.2%)	20 (18.9%)	59
FEP	S	9 (100%)	23 (79.3%)	24 (68.6%)	53 (94.6%)	50 (76.9%)	87 (82.1%)	246
	R	0 (0%)	6 (20.7%)	11 (31.4%)	3 (5.4%)	15 (23.1%)	19 (17.9%)	54
CN	S	9 (100%)	24 (82.8%)	23 (65.7%)	52 (92.9%)	50 (76.9%)	86 (81.1%)	244
	I	0 (0%)	0 (0%)	0 (0%)	1 (1.8%)	1 (1.5%)	0 (0%)	2
	R	0 (0%)	5 (17.2%)	12 (34.3%)	3 (5.4%)	14 (21.5%)	20 (18.9%)	54
CIP	S	7 (77.8%)	21 (72.4%)	19 (54.3%)	48 (85.7%)	46 (70.8%)	84 (79.2%)	225
	R	2 (22.2%)	8 (27.6%)	16 (45.7%)	8 (14.3%)	19 (29.2%)	22 (20.8%)	75
IMP	S	9 (100%)	29(100%)	35(100%)	56(100%)	65(100%)	106(100%)	300
	R	0 (0%)	0 (0%)	0 (0%)	0 (0%)	0 (0%)	0 (0%)	0
SXT	S	7 (77.8%)	21 (72.4%)	16 (45.7%)	40 (71.4%)	40 (61.5%)	70 (66%)	194
	R	0 (0%)	8 (27.6%)	17 (48.6%)	12 (21.4%)	23 (35.4%)	35 (33%)	95
ETP	S	9 (100%)	29(100%)	35(100%)	56(100%)	65(100%)	106(100%)	300
	R	0 (0%)	0 (0%)	0 (0%)	0 (0%)	0 (0%)	0 (0%)	0
LEV	S	6 (66.7%)	21 (72.4%)	18 (51.4%)	42 (75%)	39 (60%)	74 (69.8%)	200
	I	1 (11.1%)	0 (0%)	1 (2.9%)	3 (5.4%)	3 (4.6%)	0 (0%)	8
	R	2 (22.2%)	8 (27.6%)	16 (45.7%)	11 (19.6%)	23 (35.4%)	32 (30.2%)	92
NIT	S	9 (100%)	24 (82.8%)	25 (71.4%)	44 (78.6%)	54 (83.1%)	81 (76.4%)	237
	I	0 (0%)	1 (3.4%)	1 (2.9%)	0 (0%)	1 (1.5%)	1 (0.9%)	4
	R	0 (0%)	4 (13.8%)	9 (25.7%)	12 (21.4%)	10 (15.4%)	24 (22.6%)	59

Abbreviations: AMC-Amoxicillin/clavulanic acid; FEP-Cefepime; CN-Gentamicin; AMP- Ampicillin; IMP-Imipenem; CAZ-Ceftazidime; KZ-Cephazolin; CIP-Ciprofloxacin; LEV-Levofloxacin; NIT-Nitrofurantoin; CRO-Ceftriaxone; CXM-Cefuroxime; SXT-Sulfamethoxazole/trimethoprim; ETP-Ertapenem

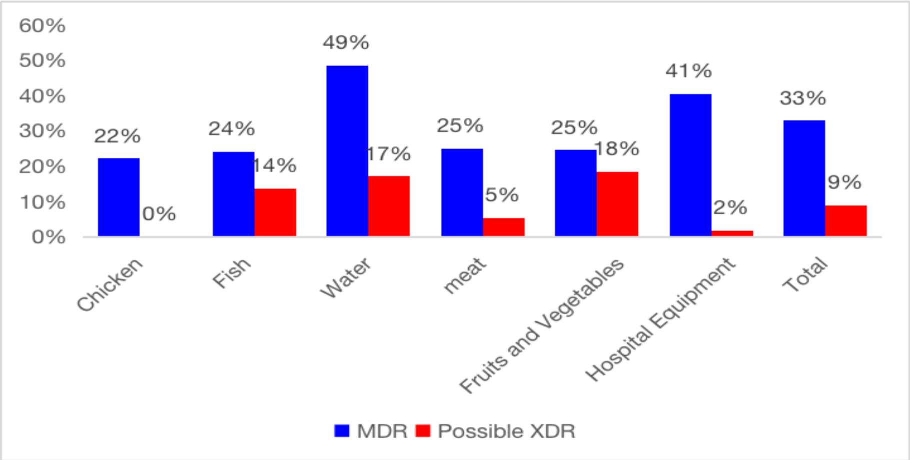

Figure S1. Distribution of MDR and XDR environmental *E. coli* isolates categorized according to sample types

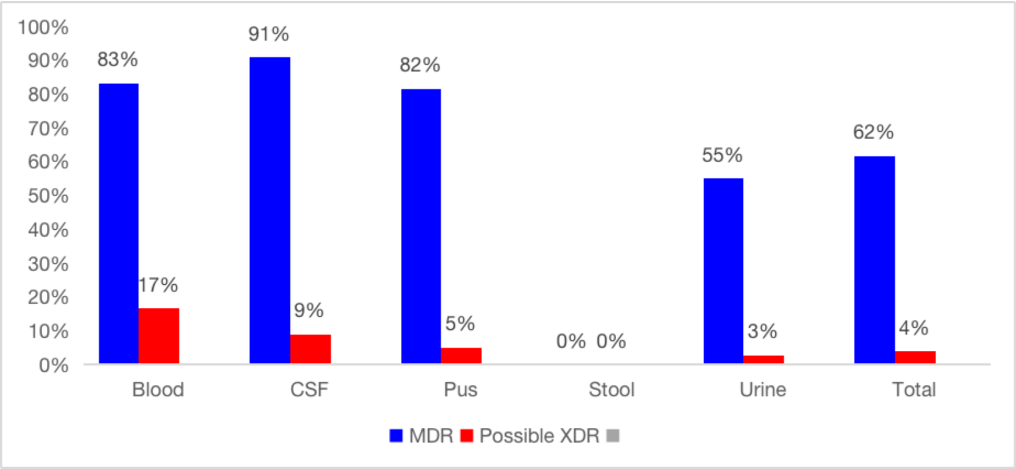

Figure S2. Distribution of MDR and XDR clinical *E. coli* isolates categorized according to sample types.

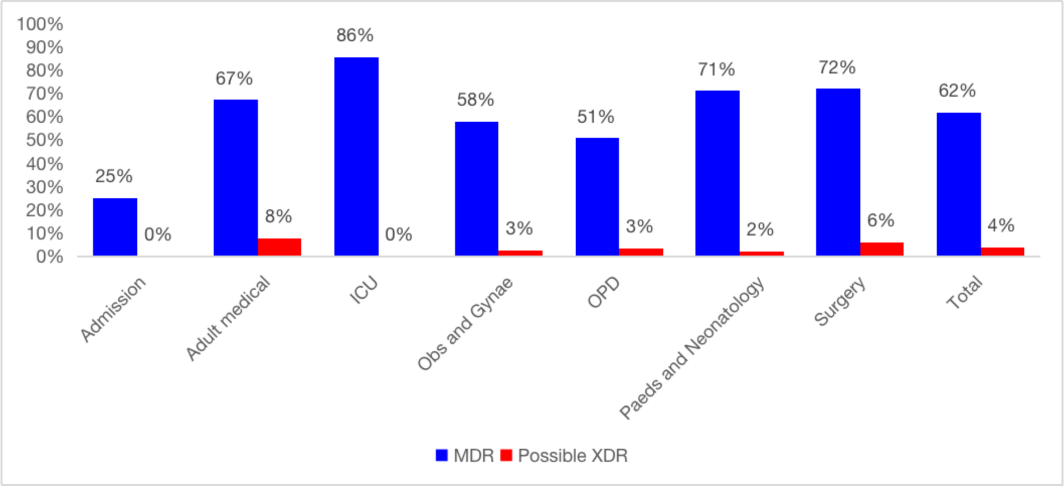

Figure S3. Distribution of MDR and XDR clinical *E. coli* isolates categorized according to hospital departments.
